# A multi-centre randomised phase III trial of Dexamethasone *vs* Dexamethasone and diethylstilbestrol in castration-resistant prostate cancer: immediate *vs* deferred Diethylstilbestrol

**DOI:** 10.1038/bjc.2011.7

**Published:** 2011-02-01

**Authors:** J Shamash, T Powles, S J Sarker, A Protheroe, N Mithal, R Mills, R Beard, P Wilson, N Tranter, N O'Brien, S McFaul, T Oliver

**Affiliations:** 1 St Bartholomew's Hospital, 7th Floor, Gloucester House, Little Britain, UK; 2 Queen Mary University London, London, UK; 3 Churchill Hospital, Oxford, UK; 4 Kent and Canterbury Hospital, Canterbury, UK; 5 Norfolk and Norwich University Hospital, Norwich, UK; 6 Worthing Hospital, Worthing, UK; 7 Queens Hospital, Romford, UK

**Keywords:** CRPC, Dexamethasone, Diethylstilbestrol, treatment sequencing

## Abstract

**Background:**

The role of further hormone therapy in castration-resistant prostate cancer (CRPC) remains unclear. We performed a multi-centre randomised phase III study comparing the use of Dexamethasone, Aspirin, and immediate addition of Diethylstilbestrol (DAiS) *vs* Dexamethasone, Aspirin, and deferred (until disease progression) addition of Diethylstilbestrol (DAdS).

**Methods:**

From 2001 to 2008, 270 men with chemotherapy-naive CRPC were randomly assigned, in a 1 : 1 ratio, to receive either DAiS or DAdS. They were stratified for performance status, presence of bone metastases, and previous normalisation of prostate-specific antigen (PSA) to androgen deprivation. The study end points were the proportion of patients achieving a 50% PSA response, progression-free survival (PFS), overall survival, and quality of life. Intention-to-treat analysis was carried out. The effect of treatment was studied first by Kaplan–Meier curves and log-rank test, and finally through multivariable stratified Cox's proportional hazards model adjusting for the effects of possible baseline prognostic factors. Quality of life was analysed using multivariate analysis of variance.

**Results:**

At study entry, the median age was 76 years (inter-quartile range: 70–80 years), the median PSA was 79 ng ml^−1^, and 76% of the cohort had metastatic disease. The response rates for DAiS (68%) and DAdS (64%) were not significantly different (*P*=0.49). Similar to the response rate, neither the PFS (median=8.1 months for both arms) nor the overall survival (19.4 *vs* 18.8 months) differed significantly between the DAiS and DAdS groups (*P*>0.20). However, the response rate for the DAiS (68%) was significantly higher than the response rate of DA (before adding Diethylstilbestrol) (50%) (*P*=0.002). Similarly, the median time to progression for DAiS (8.6 months) was significantly longer than that of DA (4.5 months) (*P*<0.001). Multivariable analysis showed that patients with previous haemoglobin ⩾11 g dl^−1^ decreased the risk of death significantly (hazard ratio: 0.44, 95% CI: 0.25–0.77). Patients treated with previous anti-androgens alone had more than 5 times more risk of death compared with patients treated with gonadorelin analogues throughout their castration-sensitive phase. Treatment sequencing did not affect the quality of life but pre-treatment performance status did. The incidence of veno–thromboembolic events was 22% (*n*=28) in DAiS and 11% (*n*=14) in the DA arm (*P*=0.02). Painful gynaecomastia occurred in only 1% on DA, whereas in 40% on DAiS (*P*=0.001).

**Conclusion:**

Dexamethasone and immediate Diethylstilbestrol resulted in neither higher PSA response rate nor higher PFS compared with Dexamethasone with deferred Diethylstilbestrol. There was no suggestion of significantly improved overall survival or quality of life. Given the significantly higher toxicity of Diethylstilbestrol, deferring Diethylstilbestrol until failure of Dexamethasone is the preferred strategy when using these agents in CRPC.

Prostate cancer is the most common cancer in men in Europe and in the United States. The frequency of patients presenting with disease has risen following the introduction of the prostate-specific antigen (PSA) blood test. The management of locally advanced or metastatic disease is initially carried out with androgen deprivation therapy, in the form of bilateral orchidectomy or gonadorelin analogues (GnRHs), with or without a peripheral anti-androgen (AA) (Hellerstedt and Pienta, 2002)) with the addition of radiotherapy to the prostate in those with locally advanced disease (Mottet *et al*, 2010; Warde *et al*, 2010). When this treatment fails, usually after 1–3 years, the disease is termed castration-resistant prostate cancer (CRPC) (Scher *et al*, 2008)), and the use of further endocrine agents and/or chemotherapy is common. Chemotherapy has been shown to improve symptoms (Tannock *et al*, 1996), and docetaxel (Petrylak *et al*, 2004; Tannock *et al*, 2004), in particular, has more recently been shown to prolong life by 2–3 months compared with mitoxantrone. The lack of robust randomised data regarding the timing and choice of further endocrine agents in this setting has resulted in no clear consensus.

A randomised study has confirmed that prednisone is superior to flutamide in terms of quality of life (Fossa *et al*, 2001). Further phase II studies have suggested that Dexamethasone results in superior PSA responses (the proportion of patients achieving a 50% reduction in PSA) (Nishimura *et al*, 2000; Venkitaraman *et al*, 2008) when compared with that observed with either prednisone/prednisolone (Tannock *et al*, 1996) or hydrocortisone (Kantoff *et al*, 1999). Whether this translates to a survival advantage over other steroids is unclear.

Oestrogens are clearly active in prostate cancer and were originally thought to function by inducing androgen deprivation indirectly. The xeno-oestrogen Diethylstilbestrol has been the most widely investigated oestrogen. More recently it has been observed that this drug is active in CRPC (Farrugia *et al*, 2000), the mechanism of this may include binding to the androgen receptor. However, Diethylstilbestrol has significant side effects, which has limited its use. These include veno–thromboembolic events (VTEs), cardiac failure, stroke, and gynaecomastia.

The combination of corticosteroids and low-dose Diethylstilbestrol (1 mg per day) is active in CRPC (Farrugia *et al*, 2000). The optimal timing of the two drug groups had not been elucidated. We therefore conducted a pragmatic randomised phase 3 trial in locally advanced or metastatic disease to see whether the drugs should be given concurrently or in sequence. As aspirin is frequently given in an attempt to reduce the risk of VTEs, yet inhibiting cyclooxygenase 2 might inhibit prostate cancer growth (Yoshimura *et al*, 2000); it was decided that those patients allocated to steroids alone would receive aspirin, to counter the criticism that any benefit of Diethylstilbestrol were in fact because of aspirin rather than to the Diethylstilbestrol.

Our objective in this trial was to compare the immediate *vs* deferred addition of Diethylstilbestrol with Dexamethasone and aspirin in treating patients with CRPC. The comparison was done in terms of response rate, time to progression, progression-free survival (PFS), overall survival, and quality of life. As patients with CRPC form a very heterogeneous group, stratified randomisation was carried out on the basis of three established factors.

## Patients and methods

### Study design

This was a multi-centre, randomised, open-label phase III trial to compare two treatment strategies in patients with newly diagnosed CRPC: the trial compared the use of Dexamethasone, Aspirin, and immediate addition of Diethylstilbestrol (DAiS) *vs* Dexamethasone, Aspirin, and deferred (until disease progression) addition of Diethylstilbestrol (DAdS). The study was approved by a multi-centre research ethics committee with local ethical approval sought by each recruiting centre. All patients gave written informed consent before enrolling in the study. Patients were randomised to the trial with a 1 : 1 ratio by stratifying according to performance status (ECOG: 0 *vs* 1–3), whether or not they achieved a PSA <4.5 ng ml^−1^ with initial androgen deprivation, and whether or not they had a positive or negative bone scan before study entry. The study was performed in accordance with the Declaration of Helsinki as well as with the Good Clinical Practice Guidelines.

### Patient population

Criteria for patients to enter the study population were as follows: they had to have a diagnosis of prostate cancer, be over 18 years old, and have an ECOG performance status 0–3. They were also required to be biochemically castrate (testosterone <1.5 nmol ml^−1^ or if not to have failed the addition of a peripheral AA), have a PSA >5 ng ml^−1^, and disease progression determined by rising PSA and/or progression of symptoms, that is, increasing pain from documented bone metastases, despite stable but elevated PSA. Patients required adequate bone marrow reserve (WBC >3 × 10^9^ per litre and platelets >50 × 10^9^ per litre) and adequate liver function (bilirubin less than 2 × upper limit of normal and ALT or AST less than 3 × upper limit of normal).

The following were exclusion criteria for the study – those with previous thrombombolic disease (including stroke), angina, and poorly controlled diabetes mellitus. Previous uncomplicated myocardial infarction was not an exclusion criterion. It was up to the individual site to decide whether or not to continue GnRH, once starting the study. Once a site had decided on their approach they were asked to maintain it for all the patients that were recruited.

### Treatment plan and outcome measures

Patients were randomly assigned to either DAiS – Dexamethasone 2 mg per day, aspirin 75 mg per day, Diethylstilbestrol 1 mg per day with ranitidine 150 mg two times per day, until symptomatic or PSA progression, or to DAdS – Dexamethasone 2 mg per day and aspirin 75 mg per day with ranitidine 150 mg two times per day. In the DAdS arm, Diethylstilbestrol was added on PSA progression that was confirmed 1 week later.

Time to PSA progression was determined using the PCWG 1 criteria (Bubley *et al*, 1999); for patients who achieved at least a 50% decrease in PSA, progression was defined as an increase in PSA of at least 50% above the nadir with an absolute increase of 5 ng ml^−1^. For those with less than 50% decrease in PSA, PSA progression was defined as an increase of at least 25% above the nadir, or in the absence of a PSA decrease, an increase of at least 25% above baseline. In either case, the increase had to be an absolute value of at least 5 ng ml^−1^. For the DAdS group, PSA response was defined if a patient responded at least once either before receiving Diethylstilbestrol or after. The use of bisphosphonates was permitted throughout the study. The time to progression (TTP) was defined as the time from randomisation to the first observation of disease progression. Progression-free survival was defined as the time from randomisation to the first observation of disease progression or death. In the DAdS group, those who received Diethylstilbestrol at the progression of disease, their TTP (PFS) was measured from randomisation to the first observation of disease progression (progression or death) after receiving Diethylstilbestrol. Survival time was measured from the randomisation into the study drug to date of death. Patients with no record of death up to the end of study were censored at their last date of assessment.

### Assessments

Patients were assessed monthly and were asked to complete a quality of life assessment as part of the study – EORTC QLQ C30 with site-specific module PR25, which specifically assessed lower urinary tract symptoms and erectile function. They were weighed at each appointment and had blood drawn for PSA, urea and electrolytes, liver function, and bone profile. Toxicity was recorded using the NCI Common Toxicity Criteria version 3.0. Patients who developed VTEs were recommended to continue hormonal therapy and start low-molecular weight heparin, and then oral anticoagulants (warfarin).

Following progression of disease, the patients were treated at the discretion of their physician or urologist. Information collected at initial assessment included haemoglobin, raised alkaline phosphatase (⩾130 IU l^−1^), previous therapy (categorised as: GnRH, AA, maximum androgen blockade (MAB), radical treatment (radiotherapy and surgery)), and comorbidities (diabetes, myocardial infarction, and respiratory disease).

### Statistical analysis

The sample size was determined to detect a clinically important difference (at least 15%) in response rate between the two study arms. On the basis of the observed response rate to Dexamethasone, 2 mg of 62% (Nishimura *et al*, 2000) and the response to Diethylstilbestrol, 1 mg with corticosteroid of 78% (Farrugia *et al*, 2000), to detect a 16% difference (62 *vs* 78%) between the two groups with 80% power, and 1 : 1 randomisation would require 130 patients in each arm (allowing for two dropouts in each arm) at 5% level of significance. This was achieved using a two-sided *χ*^2^-test (Machin *et al*, 2008)). This size of the trial also has 85% power to detect 2 months difference between median TTP according to log-rank test. Many investigators are finding that generally 5–10% change in overall quality of life score (1–100 scale) is considered significant by the patients. Therefore, it was assumed that 10% difference in overall quality of life change score between two treatment methods would be clinically important. The study would have 62% power to detect this difference with this specified sample size.

During the study, there was a period of time when the supply of Diethylstilbestrol was suspended and 12 patients required an alternative oestrogen. Therefore, an additional 12 patients were recruited to safeguard the power of the trial and an intention-to-treat analysis was performed based on all randomised patients.

Univariate analyses were performed to examine differences in baseline characteristics between the two treatment groups (DAdS *vs* DAiS) using *χ*^2^, two-sample *t*-tests, or Fisher's exact tests, as appropriate. Similarly, differences in toxicity between DA and DAiS were tested using *χ*^2^, two-sample *t*-tests, or Fisher's exact tests, as appropriate. The baseline hazard/risk of VTE was plotted using kernel density estimation, based on the DAiS group.

Univariate survival was examined using unadjusted Kaplan–Meier curves, with comparison between DAiS and DAdS by using the log-rank test. Stratified Cox proportional hazards model was applied to test the effect of treatment on overall survival adjusting for prognostic factors, including age, haemoglobin, alkaline phosphatise, previous therapy, Gleason score, and comorbidities. Pre-study androgen deprivation was highly correlated with the pre-treatment therapy. Therefore, only pre-treatment therapy was included in the multivariable Cox model. Regression diagnostics included Schoenfeld residuals. The sample was confined to complete cases for the multivariable analysis with no missing values in the considered prognostic factors. To assess selection bias because of restriction on complete cases, sensitivity analysis was performed.

Multivariate analysis of variance (MANOVA) was performed to see the effect of the two treatment approaches towards the quality of life (based on EORTC QLQ-C30) and prostate-specific quality of life (based on EORTC QLQ-PR-25). Percentage of change of scores from baseline was calculated for each repeated measurements and then they were averaged over the repetitions to apply MANOVA to test the differences in quality of life measures in terms of treatment DAdS *vs* DAiS. Intercooled STATA 10.1 (StataCorp., College Station, TX, USA) for Windows was used for the statistical analysis.

## Results

### Patient characteristics

**Figure 1:**
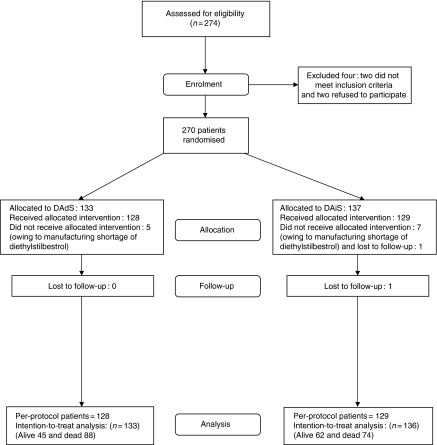
The consort flow diagram.

The patient characteristics at study entry are shown in Table 1. The median age was 76 years (inter-quartile range (IQR): 70–80) and 76% had positive bone scans. The median PSA before study entry was 79.2 ng ml^−1^ (IQR: 179 ng ml^−1^) and 39.2% had a raised (⩾130 IU l^−1^) alkaline phosphatase levels. Approximately 73% of patients had received a GnRH as their initial form of androgen deprivation. Approximately 62% of patients had previously normalised their PSA (<4.5 ng ml^−1^) to earlier androgen deprivation. The median time from starting androgen deprivation to study entry was 35 months (59 months for those with locally advanced disease and 29 months in those with metastatic disease). Among the non-metastatic group, median pre-study androgen deprivation for the DAiS group (41 months) was significantly lower than the 78 months for the DAdS group (*P*=0.02). However, this is based on only 19 patients in each group and hence is not unexpected. Approximately 47% of patients continued androgen deprivation with a GnRH. Approximately 22% of patients had previous comorbidity (previous myocardial infarction, diabetes, or chronic obstructive pulmonary disease). The study flow diagram (CONSORT) is shown in Figure 1.

### Response to therapy

The 50% PSA response to therapy was recorded according to the PCWG criteria-1 (Bubley *et al*, 1999). Overall, 68% of patients allocated to DAiS achieved a response as opposed to 64% of patients allocated to DAdS (*P*=0.49). Seven patients (2.7%) were not evaluable for response. There were two early deaths, three early withdrawals because of toxicity, and in two patients the data was insufficient to evaluate response. Similar to the response rate, neither the TTP nor the PFS differed significantly between the DAiS and DAdS groups (*P*>0.70) (Table 2). However, the response rate for the DAiS (68%) was significantly higher than the response rate of DA (before adding Diethylstilbestrol) (50%) (*P*=0.002). Similarly, the median TTP for DAiS (8.6 months) was significantly longer than that of DA (4.5 months) (*P*<0.001). The response to the addition of Diethylstilbestrol in those initially allocated to DA was 43%, with a median duration of this response of 5 months. A significant proportion of patients in this group who could have received Diethylstilbestrol did not (35%), the most common reason for this was toxicity on DA. All patients in the DAiS remained castrate throughout therapy, however 22% of patients allocated DAdS became non-castrate before the addition of Diethylstilbestrol. Their median overall time to progression was 8.4 months in this group (95% CI: 7.4–22.1) *vs* 8.3 (95% CI: 6.7–9.0) in the group that remained castrate (*P*=0.071, log-rank test).

**Figure 2:**
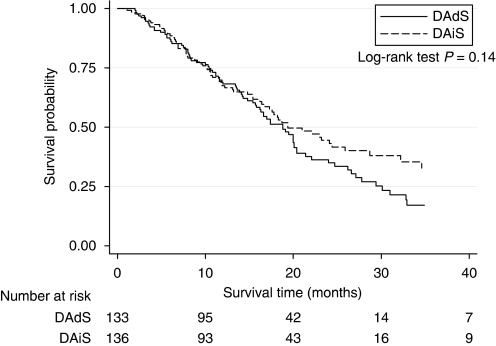
Overall survival according to immediate *vs* deferred Diethylstilbestrol.

The overall survival from entry in to the study was 19.1 months (95% CI: 16.8–21.4). There was no significant difference in overall survival when comparing the two arms; 19.4 months (95% CI: 16.8–25.9) in the DAiS arm and 18.8 months (95% CI: 15.8–20.4) in the DAdS arm. (*P*=0.28) (Figure 2).

Patients received subsequent chemotherapy at the choice of the treating physician. In the DAdS group, 30% went on to have chemotherapy and their median survival was 20.0 months (95% CI: 15.8–30.1) compared with 19.1 months (95% CI: 13.5–22.1), (*P*=0.53). In the DAiS group, 35% went on to receive chemotherapy and their median survival was 19.5 months (95% CI: 15.4–25.9) compared with 24.1 months (95% CI: 11.9–could not be estimated) (*P*=0.5).

Multivariable analysis using the Cox model (Table 3) showed that age was associated with an increased risk of death. Among other prognostic factors, having haemoglobin greater or equal to 11 g dl^−1^ decreased the risk of death significantly (HR 0.44, 95% CI: 0.25–0.77). Previous therapy had a significant effect on overall survival (*P*=0.01). Patients treated with AA alone had greater than 5 five times more risk of death compared with patients treated with GnRH. Although previous therapy and haemoglobin had significant effect on patients' overall survival, treatment sequence (DAiS *vs* DAdS) did not have any significant effect on survival when adjusted for other baseline factors (*P*=0.16). Sensitivity analysis, carried out to check for selection bias because of complete case analysis, showed that complete case analysis was unbiased.

Overall, 12 patients required oestrogen substitution because of the manufacturing shortage of Diethylstilbestrol. Therefore, the multivariable analyses were also repeated by excluding these patients, per protocol analysis, but no difference in terms of response rate, TTP and PFS, as well as in overall survival between DAiS and DAdS was found.

### Toxicity

**Figure 3:**
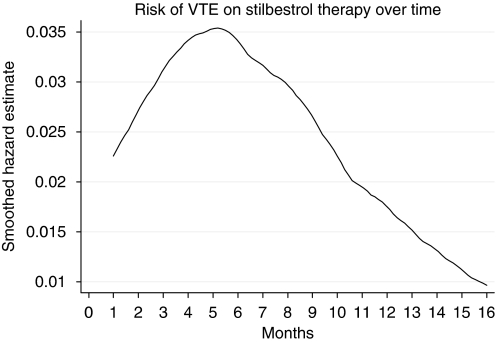
The risk of veno-thromboembolic events (VTE) from start of Diethylstilbestrol therapy over time.

The toxicity was compared between DAiS and DA (before adding Diethylstilbestrol) in Table 4. The most serious events were those due to VTE. The incidence of these was 22% (*n*=28) in DAiS and 11% (*n*=14) in the DA arm (*P*=0.02). In the DAds arm, three VTEs occurred, whilst on Dexamethasone and aspirin alone, 11 occurred following the addition of Diethylstilbestrol. One TIA occurred on DAiS. The risk of VTEs varied with time on treatment. The risk reached a maximum after 5 months of therapy and then tailed off; see Figure 3. In 60% of patients, it was possible to anticoagulate and to continue hormonal therapy.

Painful gynaecomastia occurred in only 1% on DA and in 40% on DAiS (*P*=0.001). Fluid retention was more common in DaiS, although it occurred in DA as well (49 *vs* 42%: *P*=0.26). Overall dose reductions were made in 34% of patients (39% on the DA arm and 30% on the DAiS arm *P*>0.05). No significant differences in weight gain were seen in the two arms (11% on DA and 14.0% on DAS: *P*=0.47).

### Quality of life

Multivariate analysis of variance showed no significant differences in quality of life measures of the QLQ-C30 questionnaire between the two treatment arms over time, although pre-study performance status had a significant effect on their quality of life (Table 5). However, the sample data (see Table 6) showed that the patients in DAiS group felt that they had slightly better global health and sleep compared with the patients in DAdS group. Similarly, there was no significant difference in prostate-specific symptoms of the EORTC-PR25 between the two treatment arms over the study time (MANOVA: Wilks' λ, F_5,106_=0.97, *P*<0.66). However, sample data showed that patients in DAiS group felt that wearing an incontinence aid posed more problems for them compared with the patients in DAdS group (see Table 7).

## Discussion

This study reports that the treatment strategies of immediate *vs* deferred Diethylstilbestrol do not differ significantly in terms of efficacy in the management of CRPC. Despite a slightly higher (nonsignificant) PSA response rate (68%) in the DAiS arm (with immediate use of Diethylstilbestrol), deferring its use until failure of Dexamethasone and aspirin (response rate 64%) might be the preferred strategy as PFS, overall survival, and quality of life did not reduce significantly, and there was a significantly reduced number of VTEs and painful gynaecomastia. Previous therapy and haemoglobin had significant effect on patients' overall survival rather than the treatment when adjusted for other baseline prognostic factors. Treatment sequencing did not affect the quality of life but pre-treatment performance status did. However, the response rate for the DAiS (68%) was significantly higher than the response rate of DAdS (before adding Diethylstilbestrol) (50%) (*P*=0.002). Similarly, the median TTP for DAiS (8.6 months) was significantly longer than that of DAdS (4.5 months) (*P*<0.001).

The failure to mandate continuous gonadorelin therapy throughout the trial has meant that some patients were not biochemically castrate in the DAdS arm. This could be considered as a shortcoming of the study – and in 22% of cases, testosterone recovery did occur, however this group's overall time to progression was the same as those who remained castrate, which was reassuring.

Reduction in PSA by 50% was associated with an overall survival advantage (Table 2). Recently, the importance of the reduction in PSA with chemotherapy has been questioned. Indeed, in the recently updated guidelines for reporting of Phase II studies in CRPC (Scher *et al*, 2008), the quoting of a PSA 50% reduction rate has been abandoned. It would seem that this might be premature wherein the agent concerned targets the androgen receptor as opposed to functioning independently of it.

Quality of life was assessed monthly during this study and there was no improvement conferred by the immediate use of Diethylstilbestrol. In particular, the PR25 prostate-specific questionnaire failed to show an improvement, suggesting that lower urinary tract symptoms were not helped by Diethylstilbestrol specifically. As in many studies, quality of life assessment has its limitations; there was a 26% failure to complete the forms, but this was not related to treatment (equally distributed between the two arms).

Overall, the treatments were reasonably tolerated. Diethylstilbestrol and Dexamethasone given together were associated with increased toxicity. The development of VTE was associated with the use of Diethylstilbestrol, although at this relatively low dose, the rate was manageable with no suggestion of excess mortality in this study. Moreover, many patients were able to continue Diethylstilbestrol with concomitant anticoagulation. There were no documented myocardial infarcts or development of angina on this agent.

Dexamathasone and aspirin was well tolerated with good response rates (50%). The most common toxicities were bruising and fluid retention. It is surprising that so many developed fluid retention, as Dexamethasone is considered to be a pure glucocorticoid. It is possible that the use of aspirin at the same time influenced this. Overall, 34% required dose reduction of Dexamethasone, and clearly as lower doses can produce PSA responses, starting with a smaller dose and escalating might be an alternative strategy, particularly in asymptomatic patients with rising PSA. Several groups (Nishimura *et al*, 2000; Venkitaraman *et al*, 2008), have demonstrated the value of low-dose Dexamethasone (500 m.c.g. per day) in terms of efficacy and toxicity. However, there is a suggestion of a PSA response to increasing doses (Nishimura *et al*, 2000). In patients with symptomatic disease, the improvement in appetite and weight that result from a higher dose of Dexamethasone might be more appealing, as dose escalation takes time.

When reviewed for prognostic factors, it is clear that previously identified prognostic factors from other studies apply here (Hussain *et al*, 2006). We could not identify a particular subgroup that benefitted from either treatment approach. The effect of pre-treatment haemoglobin was particularly striking with a hazard ratio of 0.44 (*P*=0.004) for overall survival, confirming the finding in many cancers that the development of anaemia is a strong adverse prognostic factor. Patients treated with AAs monotherapy fared worse, despite the fact that they needed to be biochemically castrate before study entry (via addition of GnRH analogue).

Until recently, there was a feeling that further hormone manipulations beyond androgen deprivation offered little other than transient falls in PSA, and that docetaxel-based chemotherapy should be offered for a majority following the failure of conventional androgen deprivation. It is becoming clearer that major benefits might be obtained with further hormone therapies; the interest in the drug abiraterone is supportive of this (Attard *et al*, 2009).

This study sheds some light on the immediate *vs* deferred addition of Diethylstilbestrol with Dexamethasone and Aspirin in treating patients with CRPC. Current thinking attributes prostate cancer growth and development to stimulation of the androgen receptor even at low-plasma testosterone levels (Attard *et al*, 2008). Others speculate that mutations may confer a broader range of potential ligand binding (Grigoryev *et al*, 2000; Sun *et al*, 2006), including corticosteroids and oestrogens. This hypothesis would explain the findings of this study, in that both drugs bind to the AR, but that Diethylstilbestrol has broader activity. To prove this, it would be necessary to reverse the sequence of study drugs, however, the fact that Dexamethasone has fewer severe side effects makes this unattractive.

It is possible that response to Diethylstilbestrol may have required the presence of Dexamethasone. It would have been impractical to insist that Dexamethasone be removed when Diethylstilbestrol started, in view of the fact that many would have been on it for some time making rapid withdrawal unsafe. It would have been useful to measure adrenal androgens and their precursors during the study to see whether they were related to response, and this and the addition of other translational end points would have strengthened the study. Several other shortcomings of the study were apparent. The study commenced before the docetaxel era making its usage infrequent in the study, although its use became more frequent as the study progressed. It is unclear whether this affected the outcome. In addition, the recent publication of two studies have lead to the conclusion that radiotherapy has a valuable role in the management of patients with locally advanced disease, suggesting that the number of such patients requiring systemic treatment for local progression will fall (Mottet *et al*, 2010; Warde *et al*, 2010). Disproportionate dose reduction of Dexamethasone (39% on the DA arm and 30% on the DAiS arm, *P*=0.12) might affect the study outcome slightly. Finally, as study entry required a rise in PSA, rather than progression on imaging, patients whose prostate cancer did not express PSA were not included.

In conclusion, the immediate or deferred use of Diethylstilbestrol with Dexamethasone to manage CRPC differs neither in terms of PSA response rate nor in terms of PFS. Moreover, there was no suggestion of significant difference in overall survival or in quality of life. Given the significantly higher toxicity of Diethylstilbestrol, deferring Diethylstilbestrol until failure of Dexamethasone is the preferred strategy when using these agents in CRPC. The use of hormonal agents following failure of chemotherapy for what was previously termed as hormone refractory disease is becoming more accepted (Shamash *et al*, 2008), and the use of such agents post chemotherapy warrants further investigation.

## Figures and Tables

**Table 1:** Patients' characteristics at presentation

**Patient characteristics**	**All (*N*=269)**	**DAdS (*N*=133)**	**DAiS (*N*=136)**	*P*-value^*^
Age: median (IQR), year	76 (10)	76 (11)	75.5 (10.5)	0.22
*Initial PSA:*				
Median (IQR), ng ml^−1^	79.2 (179)	76.7 (176)	81.1 (193.7)	0.34
*Pre-study androgen deprivation: median (IQR), month*				
Non-metastatic	59 (49)	78 (38)	41 (40)	0.02
Metastatic	29 (39)	34 (54)	27 (29)	0.29
				
	**No./total (%)**	**No./total (%)**	**No./total (%)**	
Metastatic disease	204/269 (76)	102/133 (77)	102/136 (75)	0.77
				
*Gleason*				0.72
⩽6	44/216 (20.4)	23/112 (20.5)	21/104 (20.2)	
7	55/216 (25.4)	26/112 (23.2)	29/104 (27.9)	
8–10	117/216 (54.2)	63/112 (56.3)	54/104 (51.9)	
				
*Initial therapy*				0.86
GNRH	188/259 (72.6)	92/130 (70.8)	96/129 (74.4)	
MAB	19/259 (7.3)	11/130 (8.4)	8/129 (6.2)	
AA	13/259 (5.0)	8/130 (6.2)	5/129 (3.9)	
Surgery	21/259 (8.1)	10/130 (7.7)	11/129 (8.5)	
RT	18/259 (7.0)	9/130 (6.9)	9/129 (7.0)	
Raised (⩾130 IU l^−1^) alkaline phosphatase	83/212 (39.2)	39/103 (37.9)	44/109 (40.4)	0.71
Haemoglobin (⩾11 g dl^−1^)	273/269 (88.1)	117/133 (88.0)	120/136 (88.2)	0.95
				
*Performance status*				1.00
0	95/269 (35.3)	47/133 (35.3)	48/136 (35.3)	
1–3	174/269 (64.7)	86/133 (64.7)	88/136 (64.7)	
Positive bone scan	204/269 (75.8)	102/133 (76.7)	102/136 (75)	0.75
PSA normalised	167/269 (62.1)	82/133 (61.7)	85/136 (62.5)	0.89
				
*Previous chronic disease*				
Myocardial infarction	12/194 (6.2)	8/101 (8)	4/93 (4.3)	0.38
Diabetes mellitus	10/194 (5.2)	3/101 (3)	7/93 (7.5)	0.20
Respiratory	23/194 (11.9)	15/101 (14.9)	8/93 (8.6)	0.18

Abbreviations: AA=anti-androgen; DAiS=Dexamethasone, Aspirin, and immediate addition of Diethylstilbestrol; DAdS=Dexamethasone, Aspirin, and deferred (until disease progression) Diethylstilbestrol; GnRH=gonadorelin analogue; IQR=inter-quartile range; MAB=maximum androgen blockade; PSA=prostate-specific antigen; RT=radiotherapy.

^*^P-values refer to differences between patients receiving DAdS and DAiS.

**Table 2:** Response to immediate (concurrent) *vs* deferred (sequential) Diethylstilbestrol

	**All (*N*=262)**	**DAdS (*N*=129)**	**DAiS (*N*=133)**	***P*-value**
PSA response, no. (%)	174 (66)	83 (64)	91 (68)	0.49
Median TTP in months (95% CI)	8.5 (7.7–10.0)	8.4 (7.6–10.4)	8.6 (5.7–10.9)	0.88
Median PFS in months (95% CI)	8.1 (7.1–8.8)	8.1 (7.4–9.0)	8.1 (5.3–10.0)	0.71
OS in months (95% CI)	19.1 (16.8–21.4)	18.8 (15.8–20.4)	19.4 (16.8–25.9)	0.28
PSA response	Median OS			
Respondent (95% CI)	26.2 (20.1–32.2)			<0.001
Non-respondent (95% CI)	11.6 (9.7–13.6)			
		DA	DAiS	
PSA response, no. (%)	174 (66)	64 (50)	91 (68)	0.002
Median TTP in months (95% CI)	5.3 (4.6–6.4)	4.5 (3.1–4.8)	8.6 (5.7–10.9)	<0.001

Abbreviations: CI=confidence interval; DA=Dexamethasone and Aspirin; DAiS=Dexamethasone, Aspirin, and immediate addition of Diethylstilbestrol; DAdS=Dexamethasone, Aspirin, and deferred (until disease progression) Diethylstilbestrol; OS=overall survival; PSA=prostate-specific antigen; TTP=time to progression.

**Table 3:** Multivariable Cox proportional hazards model for concurrent *vs* sequential treatment based on overall survival (stratified by performance status, PSA response to previous therapy and bone scan)^a^

**Characteristic**	**Hazard ratio (95% CI)^b^**	***P*-value**
Age (in years)	0.70 (0.49–1.00)	0.051
Age square	1.0025 (1.00049–1.00502)	0.046
		
*Treatment*		0.16
DAdS	1	
DAiS	0.69 (0.41–1.16)	
		
*Haemoglobin*		0.004
<11 g dl^−1^	1	
⩾11 g dl^−1^	0.44 (0.25–0.77)	
		
*Alkaline phosphatase*		0.93
<1.5 × ULN	1	
⩾1.5 × ULN	0.97 (0.55–1.71)	
		
*Gleason*		0.49
⩽6	1	
7	0.82 (0.40–1.71)	
8–10	0.68 (0.35–1.30)	
		
*Prior therapy*		0.01
GnRH	1	
AA	5.38 (1.99–14.58)	
MAB	1.91 (0.90–4.07)	
Radical treatment (radiotherapy or surgery)	1.38 (0.76–2.50)	
		
*Diabetes*		0.44
Absent	1	
Present	0.68 (0.25–1.81)	
		
*Myocardial infarction*		0.13
Absent	1	
Present	2.07 (0.81–5.27)	
		
*Respiratory disease*		0.44
Absent	1	
Present	0.73 (0.33–1.62)	

Abbreviations: AA=anti-androgen; DAiS=Dexamethasone, Aspirin, and immediate addition of Diethylstilbestrol; DAdS=Dexamethasone, Aspirin, and deferred (until disease progression) Diethylstilbestrol; CI=confidence interval; GNRH=gonadorelin analogues; MAB=maximum androgen blockade; PSA=prostate-specific antigen; ULN=upper limit of normal.

a Analysis was restricted to patients without missing values (*n*=164).

b Hazard ratios are adjusted for all variables displayed in the table.

**Table 4:** Comparison of toxicity between DA and DAiS

	**All (*N*=257)**	**DA (*N*=128)**	**DAiS (*N*=129)**	
**Toxicity**	***N* (%)**	***N* (%)**	***N* (%)**	***P*-value**
Painful gynaecomastia	52 (20)	1 (01)	51 (40)	<0.001
Headaches	28 (11)	16 (13)	12 (09)	0.31
Skin	134 (52)	59 (46)	65 (50)	0.52
Fluid retention	117 (46)	54 (42)	63 (49)	0.26
Weight gain	32 (13)	14 (11)	18 (14)	0.47
Myopathy	5 (02)	4 (03)	1 (01)	0.25
Hyperglycaemia	4 (02)	3 (02)	1 (01)	0.51
				
*VTE*	42 (16)	14 (11)	28 (22)	0.02
DVT	20 (08)	3 (03)	17 (13)	
PE	21 (08)	11 (09)	10 (08)	
TIA	1 (0.5)	0 (0)	1 (01)	

Abbreviations: DA=Dexamethasone and Aspirin; DAiS=Dexamethasone, Aspirin, and immediate addition of Diethylstilbestrol; DVT=deep venous thrombosis; PE=pulmonary embolism; TIA=transient ischaemic attack; VTE=veno–thromboembolic disease.

**Table 5:** Multivariate analysis of variance based on EOTC-QLQ-C30 in terms of immediate *vs* deferred Diethylstilbestrol and other stratifying factors

**Factor**	**Statistic**	**d.f.**	**F_(d.f.1,d.f.2)_**	***P*-value**
Treated with DAiS	0.91	1	1.01_(15, 159)_	0.44
Bad performance	0.85	1	1.83_(15, 159)_	0.03
Metastatic disease	0.87	1	1.59_(15, 159)_	0.08
PSA normalised	0.89	1	1.30_(15, 159)_	0.21

Abbreviations: DAiS=Dexamethasone, Aspirin, and immediate addition of Diethylstilbestrol; d.f.=degrees of freedom; PSA=prostate-specific antigen.

Model (Wilks λ): F_(df1, df2)_=1.55_(60, 623)_, *P*-value=0.007.

**Table 6:** Differences in quality of life scores (based on EORTC-QLQ-C30) in terms of immediate *vs* deferred Diethylstilbestrol

	**Mean % change from baseline function and symptom scores over the study period**		
**Scale**	**DAiS**	**DAdS**	**DAiS–DAdS**
Physical functioning	−0.29	−4.66	4.95
Role functioning	−1.76	−4.93	3.17
Emotional functioning	1.36	0.42	0.94
Cognitive functioning	−0.16	1.23	−1.39
Social functioning	2.79	−2.77	5.56
Global health status/QOL	0.61	−6.97	7.58
Fatigue	0.27	5.17	−4.9
Nausea and vomiting	−0.92	−0.89	−0.03
Pain	−9.46	−4.29	−5.17
Dyspnoea	5.65	9.69	−4.04
Insomnia	−6.02	0.76	−6.78
Appetite loss	−10.47	−6.12	−4.35
Constipation	−8.59	−4.73	−3.86
Diarrhoea	4.08	4.08	0
Financial difficulties	−0.14	−1.41	1.27

Abbreviations: DAiS=Dexamethasone, Aspirin, and immediate addition of Diethylstilbestrol; DAdS=Dexamethasone, Aspirin, and deferred (until disease progression) Diethylstilbestrol; QOL=quality of living.

**Table 7:** Differences in site-specific quality of life scores (based on EORTC-QLQ-PR25) in terms of immediate *vs* deferred Diethylstilbestrol

	**Mean % change from baseline scores over the study period**		
**Scale**	**DAiS**	**DAdS**	**DAiS–DAdS**
Urinary symptoms	−0.51	−0.43	−0.08
Bowel symptoms	0.25	−0.24	0.49
Hormone treatment related symptoms	0.48	−0.20	0.68
Incontinence aid	11	2.53	8.47
Sexual activity	−0.36	−0.50	0.14
Sexual function	−0.23	−0.13	−0.10

Abbreviations: DAiS=Dexamethasone, Aspirin, and immediate addition of Diethylstilbestrol, DAdS=Dexamethasone, Aspirin, and deferred (until disease progression) Diethylstilbestrol.
